# The Kitchen as the Arbiter of Human Destiny

**Published:** 1879-10

**Authors:** 


THE KITCHEN AS THE ARBITER OF HUMAN DESTINIES.—Dr. Edward Jarvis in his paper before the American Public Health Association, on "The Housekeeper's Responsibility," said:

"It depends upon the kitchen whether the family shall be robust, bright and energetic, or dull, stupid and slow. The housekeeper measures out manhood and womanhood to the family, and her position is then a highly responsible one. There is here field for the display of talent and discretion. The employments of men may offer less scope for the adaptation of great ideas to great ends than the housekeeper's. Woman is not a housekeeper by instinct or cook by nature any more than men are natural shoemakers, but she has the capacity for the former work which he may have for the latter. They cannot be perfect in the duties of a housekeeper without suitable preparation any more than they can be milliners. Boys are trained to their professions. No such training is given the daughters for that station which is admitted to be their high aim in life—the superintendence of the household. The result of carelessness in the kitchen is styled 'ill luck.' It is 'unlucky' that the bread is heavy. A carpenter might as well say it is unlucky that his window beams are too short. Nowhere except in the kitchen, where so much depends upon care in selection and preparation, is the use of the measure done away with: nowhere is so little care given to the selection of the articles of purchase. Ribbons are turned over and over to find a certain color, if some trimming for a dress is required, but the servant is sent to market to get a few pounds of any kind of beef for the table."

THE SLEEP OF PLANTS.—The phenomenon called the sleep of plants was first observed by the illustrious Swedish botanist, Linnæus. He remarked in the bird's foot trefoil the difference between the attitude of the leaves during the day and night. He almost immediately concluded that this would prove to be a general phenomenon in vegetable life. Continuing his observations, Linnæus soon satisfied himself that this change in the position of leaves during the night is observable in many vegetables. He regarded the absence of light, and not the nocturnal cold, as the principal cause of the phenomenon; for plants in hot houses close themselves during the night like those in the open air. He found the difference between waking and sleeping to be much less apparent in young plants than in more matured ones—being most clearly indicated among the compound leaves.

